# The Effect of Cow Breed and Wild Garlic Leaves (*Allium ursinum* L.) on the Sensory Quality, Volatile Compounds, and Physical Properties of Unripened Soft Rennet-Curd Cheese

**DOI:** 10.3390/foods11243948

**Published:** 2022-12-07

**Authors:** Agnieszka Pluta-Kubica, Dorota Najgebauer-Lejko, Jacek Domagała, Jana Štefániková, Jozef Golian

**Affiliations:** 1 Department of Animal Product Processing, Faculty of Food Technology, University of Agriculture in Krakow, Balicka 122, 30-149 Krakow, Poland; 2 AgroBioTech Research Centre, Slovak University of Agriculture in Nitra, Tr. A. Hlinku 2, 949 76 Nitra, Slovakia; 3 Department of Food Hygiene and Safety, Faculty of Biotechnology and Food Sciences, Slovak University of Agriculture in Nitra, Tr. A. Hlinku 2, 949 76 Nitra, Slovakia

**Keywords:** soft cheese, herbs, wild garlic, chemical composition, volatile organic compounds, flavor, color, texture

## Abstract

The aim of this study was to investigate the effects of cow breed and the addition of wild garlic on the sensory quality, volatile compounds, and physical properties of soft rennet-curd cheese. Cheese was produced from the milk of the Polish Holstein-Friesian breed Black-and-White type and the Polish Red breed, with or without the addition of wild garlic leaves. The samples were analyzed for their sensory quality, volatile compounds (using an electronic nose and GC/MS), color, and texture. The intensity of taste and smell characteristics depended only on the addition of wild garlic. PCA showed that the differences in volatile profiles resulted both from the milk cow breed and the use of wild garlic. Breed influenced almost all color parameters, while the addition of wild garlic affected all of them. The milk source, wild garlic addition, and storage duration influenced the majority of the textural parameters of the cheeses. The research conducted indicates that the addition of wild garlic leaves results in the enrichment of the volatile compound profile of cheese, making its taste and smell less milky and sour (*p* ≤ 0.001), while modifying its color and some textural properties (*p* ≤ 0.001); while, at the same time, not adversely affecting the sensory assessment of the color, appearance, texture, smell, or taste of the cheese (*p* > 0.05).

## 1. Introduction

Unripened soft rennet-curd cheese can be manufactured from the raw or pasteurized milk of various animal species: cows, sheep, and goats. Its production involves the utilization of mesophilic lactic acid bacteria (LAB) as a starter culture and rennet. Unlike mold- or smear-ripened soft rennet-curd cheese, it is consumed fresh and no maturation is required [1]. Herbs are often added to soft cheese, in order to enrich its flavor and increase variety. This is sometimes related to the traditions of the production region; e.g., traditional “Otlu cheese”, in the eastern part of Turkey, is produced with *Allium* sp., *Ferula* sp., *Tymus* sp., *Prangos* sp., *Antriscus nemorosa*, *Chaerophyllum macropodum*, *Silene vulgaris*, and *Mentha* sp. [2]. Moreover, wild garlic is added to camembert cheese for barbecuing in the Czech Republic [3]. Fresh leaves of wild garlic can also be added as a spice to flavor cottage cheese [4]. Herbs contain many aroma compounds. Moreover, their presence in cheese increases the hydrolysis of fat, which causes the release of higher levels of free fatty acids [2]. Therefore, the addition of herbs changes the profile of volatile compounds, both directly and indirectly.

Wild garlic (*Allium ursinum* L.), also known as bear garlic, ramson, or broad-leaved garlic, is used in local cuisine in Eastern Europe, as well as in Poland, Germany, the Czech Republic, and Turkey. The leaves, bulbs, and flowers of wild garlic are edible; however, its leaves have the greatest consumer use. For example, in Turkey and Poland, wild garlic leaves are used in the manufacturing of local rennet-curd cheeses [3]. Fresh soft rennet-curd cow milk cheese with wild garlic has not previously been investigated. On the other hand, researching the influence of wild garlic leaves on the properties of herby-pickled (Otlu) cheese revealed their effect on the color, determined using the CIELAB system, as well as on sensory-assessed body and texture. Nevertheless, no influence on acceptability was found [2]. Moreover, wild garlic contains many sulfur compounds. Their hydrolysis gives rise to various volatile compounds, e.g., (poly)sulfides and thiosulfinates, responsible for the specific odor and taste of this herb [4].

Cheese properties can be affected by several factors: genetic, environmental, and/or technological. The main factor among genetic aspects is breed, which indirectly affects cheese quality through its influence on milk characteristics [5]. Moreover, environmental factors are connected with feeding systems. Grazed multifloral pastures, hay, and silage fed to animals influence the milk composition in different ways. Feeding affects the basic chemical composition and sensory properties of milk, as well as its volatile profile [6]. Milk origin was found to influence the sensory and textural properties of fromage frais type cheeses. The cheese, made from Polish Red breed (RP) milk, was characterized by a more pleasant smell and lower values of hardness and chewiness than that made from Polish Holstein-Friesian breed Black-and-White type (HF) milk [7]. On the other hand, breed did not affect the color indices or firmness of camembert cheese made from Holstein and Normande cows [8].

We hypothesized that the addition of wild garlic leaves could have a positive influence on the quality of soft cow milk rennet-curd cheese. Therefore, the aim of this study was to investigate the effects of cow milk source and the addition of wild garlic leaves on the sensory quality, volatile compound profile, and physical properties of soft rennet-curd cheese.

## 2. Materials and Methods

### 2.1. Materials

Milk for production of the soft rennet-curd cheese came from two sources. Cow milk from HF was obtained directly from a farm located in Dziekanowice near Krakow (Poland), while from RP it came from the Dairy Cooperative in Bochnia (Poland). HF cows were grazed on pastures located in Dziekanowice near Krakow (Lesser Poland Voivodeship, Poland), as well as fed on freshly mown vegetation from the pasture, GMO free fodders, and hay. RP cows were grazed on fresh mountain meadows near Bochnia in the mountain and sub-mountain areas on the border of the Beskid Wyspowy mountain range and Pogórze Wiśnickie (Lesser Poland Voivodeship, Poland), as well as fed GMO free fodders and hay. The raw bovine milk was obtained in one season, the summer (from July to September).

Cheese was produced under laboratory conditions and in two independent series at the Faculty of Food Technology, University of Agriculture in Krakow, Poland. The production process was the same as previously described by Pluta-Kubica et al. [1], with minor modifications. Briefly, the milk was standardized to 2.9% fat content; pasteurized at 72 °C for 15 s; cooled down to 32 °C; enriched with anhydrous calcium chloride (0.2 g/kg of the vat milk); inoculated with a mesophilic mixed strain starter culture containing *Lactococcus lactis* subsp. *cremoris*, *Lactococcus lactis* subsp. *lactis*, *Leuconostoc mesenteroides* subsp. *cremoris,* and *Lactococcus lactis* subsp. *diacetylactis* (CHN-19, Chr. Hansen, Hørsholm, Denmark); fermented at 32 °C for 1 h; and coagulated (32 °C, 2.5 h) using a microbial rennet with activity of 2200 IMCU/g (Fromase 2200TL, Specialties, Heerlen, Denmark). Afterwards, the curd was subjected to cutting and stirring (32 °C, 0.5 h). The curd was divided into two groups before molding. Half of it was utilized for natural cheese production (N), while the remaining part was gently mixed with wild garlic leaves (5 g/kg of the curd), to obtain herbal cheese (H). Cylindrical molds with a diameter of 8 cm were used. The cheeses were drained (28 °C, 1 h; 20 °C, 18 h), brined (16% NaCl, 16–18 °C, pH 5.1–5.2, 20 min), dripped (4–6 °C, 22 h), individually packed in plastic bags, and then stored for 2 weeks at 4 °C. An average of 18.4 L and 19.3 L of milk was used to produce the HF and RP cheese, respectively. On average, 1005.0 g and 1160.8 g of HF N and H cheese was obtained, respectively. Regarding RP cheese, 1479.4 g (N) and 1539.3 g (H) of cheese were produced. The weight of cheese was determined after dripping.

The wild garlic (*Allium ursinum* L.) used in this research consisted of whole leaves (the above-ground part). Fresh material (20 kg) was collected in a privately owned forest located in the village Ropa, Lesser Poland Voivodeship, in Poland, at an altitude of 600 m above sea level. The village is located in the West Beskid Mountains, a part of the West Outer Carpathians. The leaves were harvested before the plant flowered, in early April. After picking, the fresh material was packed into a cooled box and transferred to the laboratory within 2 h. The raw material was processed on the same day. As part of preparing the raw material, the leaves were washed in cold tap water. Surface water was removed by gentle centrifugation in a leafy-vegetable centrifuge and with ambient air blowing from a fan. Any leaves that were mechanically damaged or diseased during these actions were removed. Convection drying was carried out in a ProfiLine-type chamber dryer with airflow parallel to the sieves (Hendi, The Netherlands). The charge of the material was 2 kg per 1 m^2^ of the screen. The drying temperature was 40 ± 1 °C, and the time was about 40 h, until the humidity reached 10%. Dried leaves were stored in a dry and cool place, away from light, in glass jars, and chopped using a sharp knife just before the production of cheese. The chopped leaves were 5 to 10 mm in length and width.

All reagents were of analytical grade and utilized as received, without further purification. Chemicals were produced by POCH S.A. (Gliwice, Poland), unless otherwise stated.

### 2.2. Chemical Composition, Acidity, and Water Activity Evaluation

The content of water was determined according to ISO 5534:2004 [9]. The total protein, sodium chloride, and ash contents in the cheese were analyzed according to AOAC [10]. The amount of fat was determined according to ISO 3433:2008 [11]. The acidity (pH) was electrometrically assessed using a pH-meter (CP-411, Elmetron, Poland). Water activity was measured according to ISO 18787:2017 [12] using a LabMaster-aw (Novasina AG, Lachen, Switzerland). The chemical composition of the cheese was evaluated on the day following brining. Additionally, the water content, pH, and water activity were estimated at the end of the storage period.

### 2.3. Sensory Quality Assessment

Sensory quality assessment was only performed on fresh samples (on the day following production). All cheese groups were evaluated by eight trained panelists (of age from 21 to 58) in two series (n = 64; two sources of milk × two kinds of cheese × two series of production × eight panelists). The participants were tested for their taste and smell detection thresholds, as well as ageusia and anosmia. They were familiar with the descriptive terms used and instructed about the process of evaluating the different sensory attributes. The analysis was conducted in a sensory laboratory equipped with six individual boxes. It took about 10 min per panelist. Potable water was available to the evaluators during the analysis.

The sensory evaluation of cheese samples was performed using two methods. First, a 5 point scale (1—”bad quality”, 2—”insufficient quality”, 3—”satisfactory quality”, 4—”good quality”, and 5—”very good quality”) [13] was applied to evaluate the color, external and cross-sectional appearance, texture, taste, and smell. Definitions for five quality levels for each selected trait were established (Table S1). The following indices of significance: 0.15, 0.20, 0.15, 0.25, and 0.25, respectively, were adopted. Afterwards, the overall quality was calculated (the sum of the individual scores for the properties multiplied by the corresponding indices) [13].

Second, an assessment regarding the intensity (with boundary terms from 0—‘imperceptible’, to 5—‘very intense’) of taste and smell discriminants was performed using the profiling method of quantitative descriptive analysis [14]. The following discriminants of taste were taken into account: milky, sour, herbal, bitter, piquant, salty, and pleasant; and of smell: milky, sour, herbal, and pleasant. These qualitative features were established during a special session.

### 2.4. Volatile Compound Analysis

Analysis of volatile compounds was performed using an electronic nose (e-nose) and gas chromatography -mass spectrometry (GC/MS). The samples were frozen prior to analysis, stored at −20 °C and later thawed.

The electronic nose (Heracles II, Alpha M.O.S., Toulouse, France) method employed in this study has previously been described [15,16]. Briefly, 2.5 g of the cheese sample was dynamically incubated (250 rpm) in a 20 mL vial (in a thermostat block) at 50 °C for 15 min (Autosampler, Alpha M.O.S.). Afterwards, a volume totaling of 5 mL of the headspace gaseous compounds was withdrawn using a headspace autosampler syringe and dispensed into the e-nose injector for each analysis. The selected compounds with a discriminant >0.950 were identified by matching the measured peaks using Kovats retention indices with the NIST (National Institute of Standards and Technology) library (>50%), implementing Alpha Soft V14 software (Alpha M.O.S.). The analysis was repeated three times for each sample (n = 48; two sources of milk × two kinds of cheese × two storage periods × two series of production × threefold analysis).

The head-space solid-phase microextraction (HS–SPME) method, previously described [17], was used with some modifications. Briefly, 2.5 g of sample was dynamically incubated (250 rpm) with an SPME fiber (1 cm; 50/30 µm DVB/CAR/PDMS) (Supelco, Bellefonte, PA, USA) in a 20 mL vial in a thermostat block at 50 °C for 30 min (CombiPal automated sample injector 120, CTC Analytics AG, Zwingen, Switzerland). The initial conditioning of the fiber was performed by heating the sample to 270 °C for 1 h in a SPME Fiber Cleaning and Conditioning Station (placed in the CombiPal). SPME extracts were desorbed in a GC injector at 250 °C for 1 min. Post-desorption, the fiber was cleaned at 230 °C for 10 min using the SPME Fiber Cleaning and Conditioning Station. The relative volatile profile of cheese samples was determined by GC-MS (GC 7890B–MS 5977A; Agilent Technologies Inc., Santa Clara, CA, USA), equipped with a DB–WAXms column (30 m × 0.32 mm × 0.25 µm; Agilent Technologies Inc., Santa Clara, CA, USA) and operating with a previously reported temperature program and MS conditions [17]. The identification of compounds was carried out by comparing the mass spectra (over 80% match) with a commercial database NIST^®^2017 and the Wiley library. The relative content of the determined compounds was calculated by dividing the individual peak area by the total area of all peaks. Peaks under 1% were not counted. The analysis was repeated three times for each sample (n = 48; two sources of milk × two kinds of cheese × two storage periods × two series of production × threefold analysis).

### 2.5. Analysis of Physical Properties

The cheese color was determined as previously described [18], using a Konica Minolta CM-3500d spectrophotometer (Konica Minolta Sensing Inc., Osaka, Japan) in reflectance mode under the illuminant D65/10°. The following parameters in the CIE L* a* b* system were determined: L*—lightness (from 0—”black” to 100—”white”), a* coordinate—from greenness (negative values) to redness (positive values), and b* coordinate—from blueness (negative values) to yellowness (positive values). Additionally, h°—hue angle and C*—chroma (saturation) were calculated. All cheese samples were cut into four cubes with a side length of 2 cm and the color was determined twice (n = 128; two sources of milk × two kinds of cheese × two storage periods × two series of production × four cubes × two repetitions). Each sample’s temperature was adjusted to 20 °C before conducting measurements.

Additionally, the total color difference value (Δ*E*) was calculated using the following equation (Equation (1)):

(1) $$\Delta E=\sqrt{{\left(\Delta L*\right)}^{2}+{\left(\Delta a*\right)}^{2}+{\left(\Delta b*\right)}^{2}}\;$$

where ΔL*, Δa*, and Δb* are the differences between the color value parameters of the compared cheeses. The interpretation of Δ*E* was as follows: Δ*E* < 1 means that the color differences could not be perceived by the human eye, values of Δ*E* within the range of 1–3 mean that minor color differences could be detected by the human eye, while Δ*E* > 3 means that color differences could be easily noticed by the human eye [19].

Instrumental texture profile analysis (TPA) was performed, as previously described [20], using a Universal Texture Analyser TA-XTPlus (Stable Micro Systems, Surrey, UK), controlled by a computer. Each cheese sample was cut into four cubes with a side length of 2 cm (n = 64; two sources of milk × two kinds of cheese × two storage periods × two series of production × four cubes). Their temperature was adjusted to approximately 20 °C. Compression at 60% deformation of the baseline sample height was carried out at a test speed of 1 mm/s. The test was conducted using a cylindrical compression plate 10 cm in diameter and 1 cm in height (P/100). All samples were compressed in two consecutive compression cycles. The obtained diagrams of the force dependence on time were analyzed using Texture Expert for Windows v. 1.05 (Stable Micro Systems, Surrey, UK). The Fracture TPA algorithm was applied, which allowed assignment of measures for the hardness, adhesiveness, springiness, cohesiveness, and chewiness of the cheeses.

### 2.6. Statistical Analysis

Analyses were performed in duplicate, unless otherwise stated. The obtained results, except for volatile compound determination, were statistically analyzed using Statistica version 13.3 (TIBCO Software Inc., Palo Alto, CA, USA). Means and standard deviations were calculated. Two-way ANOVA was applied for the results of the basic chemical composition and sensory quality assessment. The independent variables were milk source and wild garlic addition. Moreover, the results for water content, pH, water activity, color, and textural evaluation were subjected to three-way ANOVA. The third independent variable was storage duration. The null hypothesis was discarded for *p* ≤ 0.05 in all statistical analyses.

The results obtained during analysis of volatiles, using an e-nose (compounds with a discriminant > 0.900 were selected), were subjected to PCA (principal component analysis, Alpha M.O.S.) using Alpha Soft V14 software (Alpha M.O.S.).

## 3. Results and Discussion

### 3.1. Chemical Composition, Acidity, and Water Activity of Cheese

The basic chemical composition of the fresh cheeses is given in Table 1. A significant interaction (*p* ≤ 0.05) between the effects was only found for fat content. Milk source had a significant effect on fat (*p* ≤ 0.001) and NaCl (*p* ≤ 0.05) contents, while wild garlic addition affected the amount of protein (*p* ≤ 0.001) and ash (*p* ≤ 0.05). HF cheeses contained more fat and NaCl than RP ones. Nevertheless, the differences, although statistically significant, were somewhat small. Cheeses with wild garlic demonstrated higher protein and ash contents than cheeses without herbs. Likewise, a higher ash content due to wild garlic addition was previously reported in herby pickled cheese, and it was concluded that this herb is an important source of mineral matter [2].

The water content, acidity, and water activity of cheeses during storage is presented in Table 1. Neither milk source nor storage duration had any significant effects on the water content, pH, or water activity. The addition of wild garlic only had an influence on water content (*p* ≤ 0.001), causing its decrease. No significant interactions were found between the effects.

The decrease in water content of the cheese with wild garlic was expected. In general, dried leaves of herbs are characterized by high dry matter contents. Thus, the incorporation of wild garlic caused an increase in the dry matter content of the cheeses examined in this study. Likewise, Gliguem et al. [21] reported the higher dry matter content of double cream cheese supplemented with *Allium roseum,* in comparison to the control. Moreover, mixing the cheese curd with wild garlic leaves, although gently performed, could have promoted whey release. Nevertheless, there was no whey release during refrigerated storage.

### 3.2. Sensory Quality of Cheese

In Table 2, the results of the sensory quality assessment are presented for the fresh cheeses on a five-point scale. No significant interactions were found between effects. Milk source only had significant effects on the smell and overall quality (*p* ≤ 0.05). The smell was more desirable in the HF than in RP cheeses. Moreover, the HF cheese was characterized by a better overall quality than that made from RP milk. This was most likely a consequence of the significantly higher grades for smell appointed to the HF cheese by the panelists. The addition of wild garlic had no significant influence on the sensory features assessed using the five-point scale. Photos of the obtained cheeses are presented in Figure S1.

It is advantageous that the addition of wild garlic did not compromise the sensory quality of the cheese. Furthermore, cheese containing herbs can exhibit a rancid, too sour, too pungent, and/or unidentified bitter taste, causing low overall acceptability [22].

The discriminant intensity of the fresh cheese’s taste and smell is demonstrated in Table 2. No significant interactions were found between effects. Milk source did not affect any of the features (*p* > 0.05). On the other hand the addition of wild garlic influenced a lot of the flavor characteristics. Obviously, a herbal taste and smell were imperceptible in the natural cheeses and intense in those with wild garlic (*p* ≤ 0.001). Moreover, milky and sour tastes, as well as smell, were significantly more pronounced in the natural cheeses. Furthermore, cheeses containing wild garlic were characterized by a more intense piquant taste (*p* ≤ 0.001).

A milky and sour flavor is typical for fresh soft rennet-curd cheese. A lower intensity of these taste and smell discriminants in the H compared to N cheese could have been caused by the intense herbal flavor. Likewise a spicy hint was more pronounced in the double cream cheeses with *Allium roseum* in comparison to the plain one [21].

### 3.3. Volatile Compounds in Cheese

**Figure 1:**
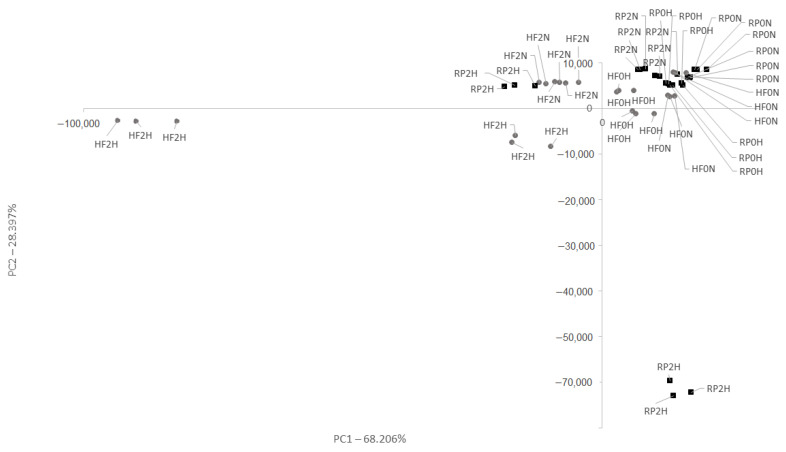
PCA of the cheese aromatic profile. Abbreviations: HF—cheese from Polish Holstein-Friesian breed Black-and-White type milk (gray); RP—cheese from Polish Red breed milk (black); 0—fresh samples; 2—samples stored for 2 weeks at 4 °C; N—cheese with no wild garlic addition; H—cheese with wild garlic.

Fresh samples of RP cheese without the addition of wild garlic (RP0N) were not significantly different (positive PC1 and PC2 axis scores) compared to the stored RP2N samples (Figure 1). On the contrary, fresh samples made from RP milk with the addition of wild garlic (RP0H) (positive PC1 and PC2 axis scores) had significantly different aroma profiles than the samples stored at 4 °C for 2 weeks. In this case, RP cheese with wild garlic made in two production series had significantly different aroma profiles following storage. The samples from the first series had an aroma profile within the positive PC1 axis score and negative PC2 axis score. In contrast, the samples from the second series had an aroma profile within the negative PC1 and positive PC2 axis scores.

Fresh samples of HF cheese without the addition of wild garlic (positive PC1 and PC2 axis scores) were significantly different from the stored ones (negative PC1 and positive PC2 axis scores). Samples of HF cheeses containing wild garlic (HF0H) had similar aromatic profiles (positive PC1 axis score 68.2%), which were significantly different from the HF2H samples of stored HF cheeses (negative PC1 and PC2 axis scores).

The volatile compounds in fresh cheese samples determined using an electronic nose and HS–SPME GC/MS are listed with their sensory descriptors in Table 3. The sensory descriptors are from the AroChemBase database (Alpha M.O.S., Toulouse, France) or The Good Scents Company Information System [23]. The obtained results enabled the indication of volatile compounds, the presence of which was related to the addition of wild garlic. These were allyl (E)-1-propenyl disulfide ((E)-1-propenyl 2-propenyl disulfide), diallyl disulfide (di-2-propenyl disulfide), and allyl methyl disulfide (methyl 2-propenyl disulfide). These sulfur compounds were previously determined in essential oils isolated from *Allium ursinum* L. leaves [4]. Moreover, other compounds, such as 3-heptanol, 3-methyl butanal, isoamyl acetate, 3-methyl butanoic acid, propanoic acid, heptanoic acid, α-pinene, and 2,3-dimethyl pyrazine were only determined in cheese samples with the addition of wild garlic. However, these volatiles are common in many types of cheeses not containing any herbs [24,25,26,27,28].

**Figure 2:**
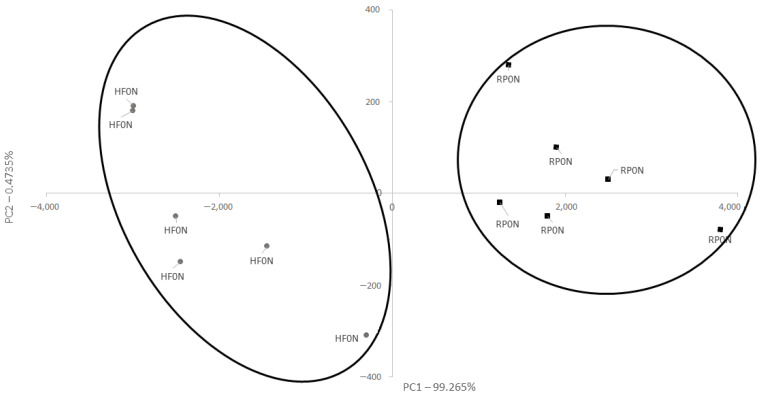
Aromatic profile PCA of fresh natural cheeses from Polish Holstein-Friesian breed Black-and-White type milk and from Polish Red breed milk. Abbreviations: HF—cheese from Polish Holstein-Friesian breed Black-and-White type milk (gray); RP—cheese from Polish Red breed milk (black); 0—fresh samples; N—cheeses with no wild garlic addition.

Significant differences (PCA) in aromatic profiles were confirmed between the fresh N cheeses made from cow milk of the RP breed (PC1 axis positive score) and the HF breed (PC1 axis negative score) (Figure 2). These profiles were different regarding the presence of four compounds: 2,3-butanediol and ethyl isobutyrate were detected in RP but absent in the HF cheese; while ethyl acetate and dihydro-2,2-dimethyl-5-phenyl-3(2H)-furanone were identified in HF but were not present in the RP cheese (Table 3).

Both the source of cow milk and storage duration had a significant influence on the volatile compound profiles in N and H cheeses. The volatile pattern of milk used for cheese manufacturing could have been one of the possible reasons for the influence of the milk source. This depends on the animal breed, age, stage of lactation, and diet (which was different for the HF and RP cows, although the research was conducted during the same season) [29,30]. Moreover, storage duration typically influences the volatile profile in dairy products, such as e.g., fresh goat cheese and yoghurt [31].

### 3.4. Physical Properties of Cheese

The color parameters of the fresh and stored cheeses are presented in Table 4. Milk source influenced almost all of them (*p* ≤ 0.001), with the exception of L*. The addition of wild garlic affected all parameters (*p* ≤ 0.001). L* and a* values were stable during storage; however, the b* and C* values increased (*p* ≤ 0.001), while the hue angle decreased (*p* ≤ 0.01), after two weeks of refrigeration.

HF cheeses were more greenish and less yellowish than the RP ones. The red color coordinate prevailed over the green one in RP cheeses without wild garlic. Moreover, HF cheeses were characterized by a higher hue angle of approximately 90.58–100.07°, which is between the range of yellow (90°) and green (180°). Wild garlic addition triggered a lowering of L* values and made the cheeses more greenish and yellowish. Likewise, a decrease of L* and increase in the green color saturation were observed in a pickled cheese, due to herb addition [2].

RP cheeses had a higher color saturation intensity than HF ones, and the addition of wild garlic was associated with an increase in the value of this parameter. The chroma value also increased during the storage of cheeses. These differences were statistically significant; however, taking the general C* range from 0 to 60 into, they were somewhat small.

The calculation of Δ*E* demonstrated that the fresh N HF cheeses showed a clear color difference when compared to the fresh N RP samples (Δ*E* = 3.64). Notwithstanding, the total color difference between the cheeses after storage was within the range of 1–3 (Δ*E* = 2.39), meaning that only minor color differences could be detected by the human eye. Similarly, the comparison between the fresh and stored HF cheeses (separately for N and H) revealed that the Δ*E* was between 1 and 3 (2.09 and 1.46, respectively). In comparison, storage did not trigger a noticeable color change in N and H RP cheeses (Δ*E* equaled 0.98 and 0.66, respectively).

The textural parameters of the fresh and stored cheeses are presented in Table 4. Milk source and wild garlic addition affected the hardness and adhesiveness (*p* ≤ 0.001), as well as the chewiness (*p* ≤ 0.001 and *p* ≤ 0.05, respectively). Furthermore, the addition of herbs influenced the springiness of cheeses (*p* ≤ 0.001). The hardness and adhesiveness did not change during the storage period; while the springiness, cohesiveness, and chewiness experienced a decrease (*p* ≤ 0.05, *p* ≤ 0.001 and *p* ≤ 0.001, respectively).

HF cheeses were characterized by a lower hardness, adhesiveness, and chewiness than the RP ones. The addition of wild garlic resulted in an increase in hardness and chewiness. It also triggered a decrease in the adhesiveness and springiness.

A higher hardness noted in RP cheese, compared to HF, was probably caused by their lower fat content (Table 1). In general, fat reduction is related to an increase in hardness [32]. Moreover, the lower hardness of the N compared to H cheeses was most likely influenced by the water content, which was higher in the N cheeses (Table 1), making them softer. Likewise, the lower springiness value of the H cheeses in comparison to those from the N group was probably caused by the lower water content. H cheese samples crumbled during TPA. Differences in the chewiness values were the result of multiplying the hardness by springiness and by the cohesiveness [32].

## 4. Conclusions

To the best of our knowledge, fresh soft rennet-curd cow milk cheese with wild garlic leaves has not previously been investigated. The research conducted allowed demonstrating that the source of cow milk (cow breed), wild garlic addition, and storage duration significantly influenced various cheese characteristics. The addition of wild garlic leaves has a positive influence on the quality of soft cow milk rennet-curd cheese. It results in the enrichment of the volatile compound profile of cheese, making its taste and smell less milky and sour, while modifying its color and textural properties, which, at the same time, does not adversely affect the sensory assessment of the color, appearance, texture, smell, or taste of cheese with herbs. Therefore, wild garlic leaves can be recommended as an additive in the production of soft cow milk rennet-curd cheese, regardless of the cow milk source (HF or RP).

## Figures and Tables

**Table 1:** Basic chemical composition, acidity, and water activity of cheeses (mean ± SD).

Feature	Storage Time (Weeks)	Milk Source				*p*-Value		
		HF		RP				
		N	H	N	H	S	A	T
Water content (%)	0	53.5 ± 1.3	47.2 ± 1.9	58.2 ± 2.3	51.5 ± 3.0	NS	***	NS
	2	56.3 ± 1.3	47.1 ± 10.1	57.4 ± 1.8	48.5 ± 3.7			
pH	0	4.5 ± 0.2	4.6 ± 0.2	4.7 ± 0.1	4.5 ± 0.0	NS	NS	NS
	2	4.8 ± 0.6	4.4 ± 0.2	4.5 ± 0.1	4.6 ± 0.1			
Water activity	0	0.95 ± 0.03	0.94 ± 0.02	0.92 ± 0.00	0.93 ± 0.02	NS	NS	NS
	2	0.94 ± 0.02	0.94 ± 0.02	0.95 ± 0.03	0.95 ± 0.03			
Fat content (%)	0	24.8 ± 2.1	26.4 ± 2.5	24.1 ± 1.8	21.1 ± 0.9	***	NS	ne
Protein content (%)	0	18.8 ± 1.6	23.4 ± 1.9	18.5 ± 2.3	21.2 ± 1.5	NS	***	ne
Ash content (%)	0	2.32 ± 0.27	2.63 ± 0.31	2.23 ± 0.05	2.57 ± 0.23	NS	*	ne
NaCl content (%)	0	0.6 ± 0.2	0.7 ± 0.1	0.5 ± 0.1	0.4 ± 0.2	*	NS	ne

Abbreviations: HF—cheese from Polish Holstein-Friesian breed Black-and-White type milk; RP—cheese from Polish Red breed milk; 0—fresh samples; 2—samples stored for 2 weeks at 4 °C; N—cheese with no wild garlic addition; H—cheese with wild garlic; S—breed effect; A—wild garlic addition effect; T—storage duration effect; ne—not examined. * *p* ≤ 0.05; *** *p* ≤ 0.001; NS: *p* > 0.05. Significant interaction (*p* ≤ 0.05): S×A for fat content. Values are expressed as the mean of four determinations (two series of production × two repetitions) ± standard deviation (SD).

**Table 2:** Sensory quality of cheeses (mean ± SD).

Feature	Milk Source				*p*-Value	
	HF		RP			
	N	H	N	H	S	A
Sensory quality of cheeses on a five-point scale						
Color	4.91 ± 0.20	4.81 ± 0.36	4.63 ± 0.62	4.66 ± 0.60	NS	NS
Appearance	4.56 ± 0.54	4.69 ± 0.48	4.41 ± 0.61	4.34 ± 0.57	NS	NS
Texture	4.53 ± 0.62	4.63 ± 0.43	4.38 ± 0.56	4.31 ± 0.44	NS	NS
Taste	4.59 ± 0.46	4.66 ± 0.44	4.53 ± 0.62	4.66 ± 0.44	NS	NS
Smell	4.75 ± 0.37	4.84 ± 0.35	4.41 ± 0.61	4.53 ± 0.59	*	NS
Overall quality	4.66 ± 0.27	4.73 ± 0.23	4.47 ± 0.43	4.51 ± 0.33	*	NS
Discriminant intensity of cheese taste and smell						
Milky taste	3.25 ± 1.39	2.47 ± 1.77	3.19 ± 1.11	2.06 ± 1.06	NS	***
Sour taste	2.81 ± 1.11	2.09 ± 1.10	3.25 ± 1.00	2.31 ± 0.95	NS	***
Herbal taste	0.00 ± 0.00	4.19 ± 0.68	0.00 ± 0.00	4.06 ± 0.93	NS	***
Bitter taste	0.47 ± 0.88	0.75 ± 0.68	0.63 ± 1.02	1.00 ± 1.03	NS	NS
Piquant taste	0.31 ± 0.60	2.22 ± 1.48	0.50 ± 0.89	1.94 ± 1.53	NS	***
Salty taste	2.94 ± 0.98	2.69 ± 0.95	2.56 ± 0.96	2.56 ± 0.89	NS	NS
Pleasant taste	4.44 ± 0.73	4.47 ± 0.56	4.25 ± 0.77	4.19 ± 0.66	NS	NS
Milky smell	3.25 ± 1.34	1.94 ± 1.69	3.31 ± 1.01	1.94 ± 1.18	NS	***
Sour smell	2.50 ± 1.21	2.16 ± 1.21	2.81 ± 1.38	1.81 ± 1.11	NS	*
Herbal smell	0.00 ± 0.00	4.41 ± 0.71	0.00 ± 0.00	4.25 ± 1.18	NS	***
Pleasant smell	4.53 ± 0.62	4.44 ± 0.81	4.19 ± 0.83	4.44 ± 0.73	NS	NS

Abbreviations: HF—cheese from Polish Holstein-Friesian breed Black-and-White type milk; RP—cheese from Polish Red breed milk; N—cheese with no wild garlic addition; H—cheese with wild garlic; S—breed effect; A—wild garlic addition effect. * *p* ≤ 0.05; *** *p* ≤ 0.001; NS: *p* > 0.05. No significant interactions were found between effects (*p* > 0.05). Values are expressed as the mean of sixteen determinations (two series of production × eight panelists) ± standard deviation (SD).

**Table 3:** Volatile organic compounds in fresh cheese samples determined using an electronic nose with D > 0.9500 and via HS–SPME GC/MS.

Compounds		Determination Method	Sensory Descriptors ^1^	HF N	HF H	RP N	RP H
Alcohols	2-propanol	e-nose	alcoholic, ethereal	+	+	+	+
	2-methyl-propanol	e-nose	alcoholic, bitter, chemical, glue, leek, licorice, solvent	+	+	+	+
	2,3-butanediol	HS–SPME GC/MS	fruity, onion			+	
	3-heptanol	e-nose	green, herbaceous		+		+
Aldehydes	acetaldehyde	e-nose	ethereal, fresh, fruity, pungent	+	+	+	+
	propanal	e-nose	ethereal, plastic, pungent, solvent	+	+	+	+
	2-methyl propanal	e-nose	burnt, fruity, green, malty, pungent, spicy, toasted	+	+	+	+
	3-methyl butanal	e-nose	almond, fruity, green, herbaceous, malty, toasted		+		
	benzaldehyde	HS–SPME GC/MS	almond, burnt sugar, fruity, woody	+		+	
Ketones	2,3-butanedione	e-nose and HS–SPME GC/MS	butter, caramelized, creamy, fruity, pineapple, spirit	+/+	+/+	+/+	+/+
	3-hydroxy-2-butanone	HS–SPME GC/MS	sweet buttery creamy, dairy, milky, fatty	+	+	+	+
Esters	ethyl acetate	e-nose	acidic, caramelized, fruity, pineapple, solvent, butter, ethereal, orange, pungent, sweet	+	+		+
	ethyl acrylate	e-nose	fruity	+	+	+	+
	ethyl isobutyrate	e-nose	fruity, rubber, strawberry, sweet		+	+	+
	ethyl propanoate	e-nose	acetone, fruity, solvent	+	+	+	+
	isoamyl acetate	e-nose	banana, fresh, fruity, pear, sweet		+		+
	α-terpineol acetate	HS–SPME GC/MS	-	+		+	
Free fatty acids	2-methyl propanoic acid	e-nose	acidic, butter, cheese, fatty, phenolic, rancid, sweaty	+	+	+	+
	3-methyl butanoic acid	e-nose	acidic, cheese, rancid, sweaty		+		+
	acetic acid	e-nose and HS–SPME GC/MS	acidic, pungent, sour, vinegar	+	+/+	+/+	+/+
	propanoic acid	e-nose	acidic, pungent, rancid, soy				+
	butanoic acid	e-nose and HS–SPME GC/MS	butter, cheese, rancid, sweaty	+/+	+/+	+/+	+/+
	hexanoic acid	HS–SPME GC/MS	cheese, fatty, goat, pungent, rancid, sweaty	+	+	+	+
	heptanoic acid	HS–SPME GC/MS	cheese, fatty, rancid, sour-sweat				+
	octanoic acid	HS–SPME GC/MS	cheese, fatty, fatty acid, fresh, mossy, sweaty	+	+	+	+
Sulfur compounds	allyl (E)-1-propenyl disulfide	HS–SPME GC/MS	sulfurous, alliaceous		+		+
	diallyl disulfide	HS–SPME GC/MS	alliaceous, onion, garlic, metallic		+		+
	allyl methyl disulfide	HS–SPME GC/MS	alliaceous, onion, garlic, green onion		+		+
Terpenes	D-limonene	HS–SPME GC/MS	citrus, fruity, minty, orange, peely	+		+	+
	α-pinene	e-nose	pine, terpenic		+		+
Furans	dihydro-2,2-dimethyl-5-phenyl-3(2H)-furanone	HS–SPME GC/MS	-	+	+		+
Pyrazines	2,3-dimethyl pyrazine	e-nose	baked, cocoa, coffee, nutty, caramelized, meaty, peanut, butter		+		+
Hydrocarbons	6-methyl-octadecane	HS–SPME GC/MS	-	+		+	
Oxime	methoxy-phenyl-oxime	HS–SPME GC/MS	-	+	+	+	+

^1^ Sensory descriptors are from the AroChemBase database (Alpha M.O.S., Toulouse, France) or The Good Scents Company Information System [23]. Abbreviations: HF—cheese from Polish Holstein-Friesian breed Black-and-White type milk; RP—cheese from Polish Red breed milk; N—cheese with no wild garlic addition; H—cheese with wild garlic. “+” means that the compound was detected.

**Table 4:** Color and textural parameters of the cheeses (mean ± SD).

Feature	Storage Duration (Weeks)	Milk Source				*p*-Value		
		HF		RP				
		N	H	N	H	S	A	T
Color parameters								
L*	0	85.43 ± 3.04	74.10 ± 5.23	83.46 ± 2.59	73.92 ± 6.32	NS	***	NS
	2	83.68 ± 1.79	73.78 ± 7.53	84.38 ± 2.05	73.82 ± 5.17			
a*	0	−0.26 ± 0.45	−2.19 ± 0.67	0.63 ± 0.20	−1.06 ± 0.63	***	***	NS
	2	−0.09 ± 0.40	−1.79 ± 0.77	0.67 ± 0.15	−1.00 ± 0.44			
b*	0	11.78 ± 2.44	13.23 ± 2.38	14.71 ± 1.61	15.34 ± 1.66	***	***	***
	2	12.91 ± 1.59	14.59 ± 2.02	15.06 ± 1.53	15.99 ± 1.25			
h	0	91.72 ± 2.45	100.07 ± 4.35	87.64 ± 0.64	94.07 ± 2.53	***	***	**
	2	90.58 ± 1.86	97.38 ± 3.85	87.55 ± 0.54	93.62 ± 1.57			
C*	0	11.79 ± 2.42	13.44 ± 2.25	14.71 ± 1.61	15.39 ± 1.63	***	***	***
	2	12.91 ± 1.59	14.73 ± 1.94	15.07 ± 1.53	16.03 ± 1.23			
Textural parameters								
Hardness (kG)	0	2.18 ± 0.21	3.51 ± 0.50	3.06 ± 0.71	4.40 ± 0.58	***	***	NS
	2	2.14 ± 0.37	2.96 ± 0.47	3.07 ± 0.44	4.08 ± 0.39			
Adhesiveness (|kG × s|)	0	0.12 ± 0.05	0.07 ± 0.05	0.13 ± 0.08	0.12 ± 0.08	***	***	NS
	2	0.12 ± 0.03	0.11 ± 0.06	0.21 ± 0.10	0.13 ± 0.06			
Springiness (-)	0	0.79 ± 0.06	0.61 ± 0.04	0.65 ± 0.16	0.59 ± 0.12	NS	***	*
	2	0.64 ± 0.08	0.55 ± 0.05	0.71 ± 0.07	0.53 ± 0.05			
Cohesiveness (-)	0	0.23 ± 0.03	0.21 ± 0.02	0.24 ± 0.12	0.25 ± 0.12	NS	NS	***
	2	0.19 ± 0.02	0.17 ± 0.01	0.18 ± 0.01	0.16 ± 0.04			
Chewiness (kG)	0	0.40 ± 0.05	0.44 ± 0.09	0.44 ± 0.14	0.60 ± 0.13	***	*	***
	2	0.25 ± 0.07	0.28 ± 0.06	0.38 ± 0.07	0.34 ± 0.06			

Abbreviations: HF—cheese from Polish Holstein-Friesian breed Black-and-White type milk; RP—cheese from Polish Red breed milk; 0—fresh samples; 2—samples stored for 2 weeks at 4 °C; N—cheese with no wild garlic addition; H—cheese with wild garlic; S—breed effect; A—wild garlic addition effect; T—storage duration effect. * *p* ≤ 0.05; ** *p* ≤ 0.01; *** *p* ≤ 0.001; NS: *p* > 0.05. Significant interactions (*p* ≤ 0.05): S×T and S×A×T for springiness, A×T for chewiness. Values of color parameters are expressed as the mean of sixteen determinations (two series of production × four cubes × two repetitions) ± standard deviation (SD). Values of textural parameters are expressed as the mean of eight determinations (two series of production × four cubes) ± standard deviation (SD).

## Data Availability

The data used to support the findings of this study can be made available by the corresponding author upon request.

## Supplementary Materials

The following are available online at https://www.mdpi.com/article/10.3390/foods11243948/s1, Figure S1. The photos of natural and herbal cheese during molding (A) and during dripping after brining (B), Table S1. The definitions of five quality levels for each selected trait in sensory analysis using a 5-point scale.

## References

[1] Pluta-Kubica A., Jamróz E., Kawecka A., Juszczak L., Krzyściak P. (2020). Active Edible Furcellaran/Whey Protein Films with Yerba Mate and White Tea Extracts: Preparation, Characterization and Its Application to Fresh Soft Rennet-Curd Cheese. Int. J. Biol. Macromol..

[2] Tarakci Z., Temiz H., Aykut U., Turhan S. (2011). Influence of Wild Garlic on Color, Free Fatty Acids, and Chemical and Sensory Properties of Herby Pickled Cheese. Int. J. Food Prop..

[3] Gębczyński P., Bernaś E., Słupski J., Hernik J., Walczycka M., Sankowski E., Harris B.J. (2022). Usage of Wild-Growing Plants as Foodstuff. Cultural Heritage—Possibilities for Land-centered Societal Development.

[4] Sobolewska D., Podolak I., Makowska-Wąs J. (2015). *Allium ursinum*: Botanical, Phytochemical and Pharmacological Overview. Phytochem. Rev..

[5] De Marchi M., Bittante G., Dal Zotto R., Dalvit C., Cassandro M. (2008). Effect of Holstein Friesian and Brown Swiss Breeds on Quality of Milk and Cheese. J. Dairy Sci..

[6] Kalač P. (2011). The Effects of Silage Feeding on Some Sensory and Health Attributes of Cow’s Milk: A Review. Food Chem..

[7] Domagała J., Pluta-Kubica A., Sady M., Bonczar G., Duda I., Pustkowiak H. (2020). Comparison of the Composition and Quality Properties of Fromage Frais-Type Cheese Manufactured from the Milk of Selected Cow Breeds. Ann. Anim. Sci..

[8] Hurtaud C., Peyraud J.L., Michel G., Berthelot D., Delaby L. (2009). Winter Feeding Systems and Dairy Cow Breed Have an Impact on Milk Composition and Flavour of Two Protected Designation of Origin French Cheeses. Animal.

[9] (2004). Cheese and Processed Cheese—Determination of the Total Solids Content.

[10] AOAC (2007). Official Methods of Analysis of AOAC International.

[11] (2008). Cheese–Determination of Fat Content–Van Gulik Method.

[12] (2017). Foodstuffs—Determination of Water Activity.

[13] Gawęcka J., Jędryka T. (2001). Rozdział 5. Metody Punktowe. Analiza Sensoryczna: Wybrane Metody i Przykłady Zastosowań.

[14] Baryłko-Pikielna N., Matuszewska I. (2014). Rozdział 10. Metody Sensorycznej Analizy Opisowej. Sensoryczne Badania Żywności. Podstawy-Metody-Zastosowania.

[15] Štefániková J., Ducková V., Miškeje M., Kacániová M., Canigová M. (2020). The Impact of Different Factors on the Quality and Volatile Organic Compounds Profile in “Bryndza” Cheese. Foods.

[16] Štefániková J., Nagyová V., Hynšt M., Vietoris V., Martišová P., Nagyová Ľ. (2019). Application of Electronic Nose for Determination of Slovak Cheese Authentication Based on Aroma Profile. Potravin. Slovak J. Food Sci..

[17] Sádecká J., Kolek E., Pangallo D., Valík L., Kuchta T. (2014). Principal Volatile Odorants and Dynamics of Their Formation during the Production of May Bryndza Cheese. Food Chem..

[18] Najgebauer-Lejko D., Liszka K., Tabaszewska M., Domagała J. (2021). Probiotic Yoghurts with Sea Buckthorn, Elderberry, and Sloe Fruit Purees. Molecules.

[19] Quintanilla P., Beltrán M.C., Molina A., Escriche I., Molina M.P. (2019). Characteristics of Ripened Tronchón Cheese from Raw Goat Milk Containing Legally Admissible Amounts of Antibiotics. J. Dairy Sci..

[20] Filipczak-Fiutak M., Pluta-Kubica A., Domagała J., Duda I., Migdał W. (2021). Nutritional Value and Organoleptic Assessment of Traditionally Smoked Cheeses Made from Goat, Sheep and Cow’s Milk. PLoS ONE.

[21] Gliguem H., Ben Hassine D., Ben Haj Said L., Ben Tekaya I., Rahmani R., Bellagha S. (2021). Supplementation of Double Cream Cheese with Allium Roseum: Effects on Quality Improvement and Shelf-Life Extension. Foods.

[22] Güler Z. (2014). Profiles of Organic Acid and Volatile Compounds in Acid-Type Cheeses Containing Herbs and Spices (Surk Cheese). Int. J. Food Prop..

[23] The Good Scents Company Information System [WWW Document], n.d. Http://Www.Thegoodscentscompany.Com/.

[24] Pluta-Kubica A., Domagała J., Gąsior R., Wojtycza K., Witczak M. (2021). Characterisation of the Profile of Volatiles of Polish Emmental Cheese. Int. Dairy J..

[25] Pillonel L., Ampuero S., Tabacchi R., Bosset J.O. (2003). Analytical Methods for the Determination of the Geographic Origin of Emmental Cheese: Volatile Compounds by GC/MS-FID and Electronic Nose. Eur. Food Res. Technol..

[26] Frank D.C., Owen C.M., Patterson J. (2004). Solid Phase Microextraction (SPME) Combined with Gas-Chromatography and Olfactometry-Mass Spectrometry for Characterization of Cheese Aroma Compounds. LWT-Food Sci. Technol..

[27] Marilley L., Casey M.G. (2004). Flavours of Cheese Products: Metabolic Pathways, Analytical Tools and Identification of Producing Strains. Int. J. Food Microbiol..

[28] Endrizzi I., Fabris A., Biasioli F., Aprea E., Franciosi E., Poznanski E., Cavazza A., Gasperi F. (2012). The Effect of Milk Collection and Storage Conditions on the Final Quality of Trentingrana Cheese: Sensory and Instrumental Evaluation. Int. Dairy J..

[29] Foroutan A., Guo A.C., Vazquez-Fresno R., Lipfert M., Zhang L., Zheng J., Badran H., Budinski Z., Mandal R., Ametaj B.N. (2019). Chemical Composition of Commercial Cow’s Milk. J. Agric. Food Chem..

[30] Mordenti A.L., Brogna N., Formigoni A. (2017). REVIEW: The Link between Feeding Dairy Cows and Parmigiano-Reggiano Cheese Production Area. Prof. Anim. Sci..

[31] Condurso C., Verzera A., Romeo V., Ziino M., Conte F. (2008). Solid-Phase Microextraction and Gas Chromatography Mass Spectrometry Analysis of Dairy Product Volatiles for the Determination of Shelf-Life. Int. Dairy J..

[32] Henneberry S., Wilkinson M.G., Kilcawley K.N., Kelly P.M., Guinee T.P. (2015). Interactive Effects of Salt and Fat Reduction on Composition, Rheology and Functional Properties of Mozzarella-Style Cheese. Dairy Sci. Technol..

