# Supplementary material for: The Effect of Cow Breed and Wild Garlic Leaves (Allium ursinum L.) on the Sensory Quality, Volatile Compounds, and Physical Properties of Unripened Soft Rennet-Curd Cheese
Source: Foods. 2022 Dec 7;11(24):3948. doi: 10.3390/foods11243948 (PMC9778303; doi:10.3390/foods11243948)

Supplementary material

Table S1. The definitions of five quality levels for each selected trait in sensory analysis using a 5-point scale

Traits	5	4	3	2	1
Color	White, slightly creamy color or characteristic of the herbal additive	White, slightly creamy color with slight discoloration	Creamy color with slight discoloration, slightly uneven	Cream color with a tint of yellow, uneven	Yellow to brown, very uneven
External and cross-sectional appearance	Uniform cheese, smooth, without lumps, evenly distributed herbal additive	Delicate cracks, occasionally uneven cheese	Cracks, uneven cheese, occasional lumps	Clearly uneven cheese, visible lumps	Uneven, cracked cheese, visible lumps, unevenly distributed herbal additive
Texture	Firm cheese, no lumps	Firm cheese, slight cracks visible, slightly crumbly, slightly greasy	Greasy, crumbly, cracked cheese	Dry, hard or too soft and greasy cheese	Very hard or very soft, very cracked, visible lumps
Taste	Clean, very pleasant, slightly spicy. Perceptible taste of herbal additive	Pleasant, clean, slightly perceptible taste of the herbal additive	Strong, spicy or tasteless	Strong, spicy, noticeable foreign flavors, bitter, slightly rancid	Very unpleasant, foreign, very bitter, very sour, rancid
Smell	Very pleasant, clean. Perceptible smell of herbal additive	Pleasant, clean, slightly perceptible smell of herbal additive	Sharp, slightly foreign or odorless	Foreign, spicy, stale, slightly rancid	Unpleasant, clearly stale, rancid

Figure S1. The photos of natural and herbal cheese during molding (A) and during dripping after brining (B)

A

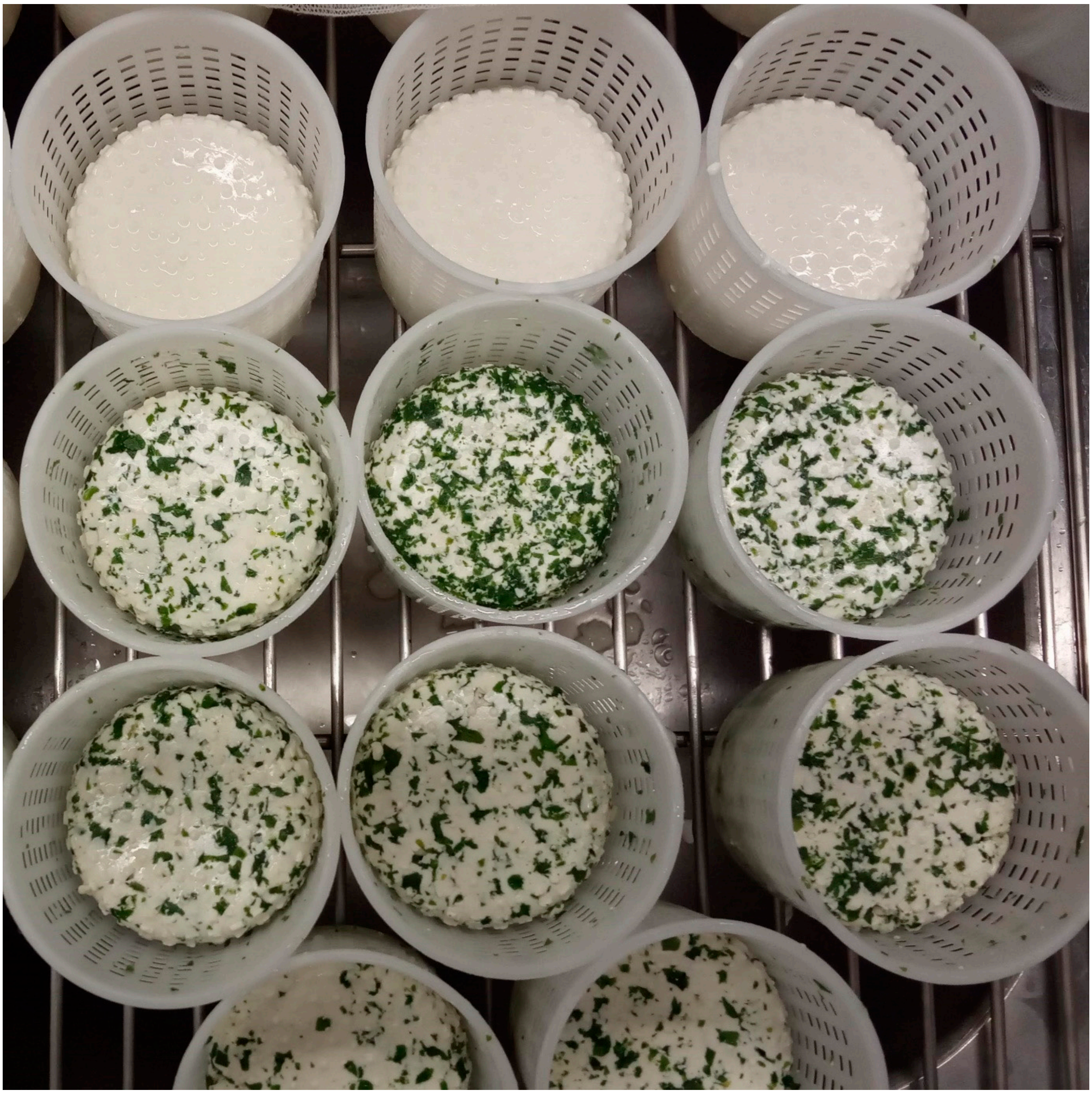

B

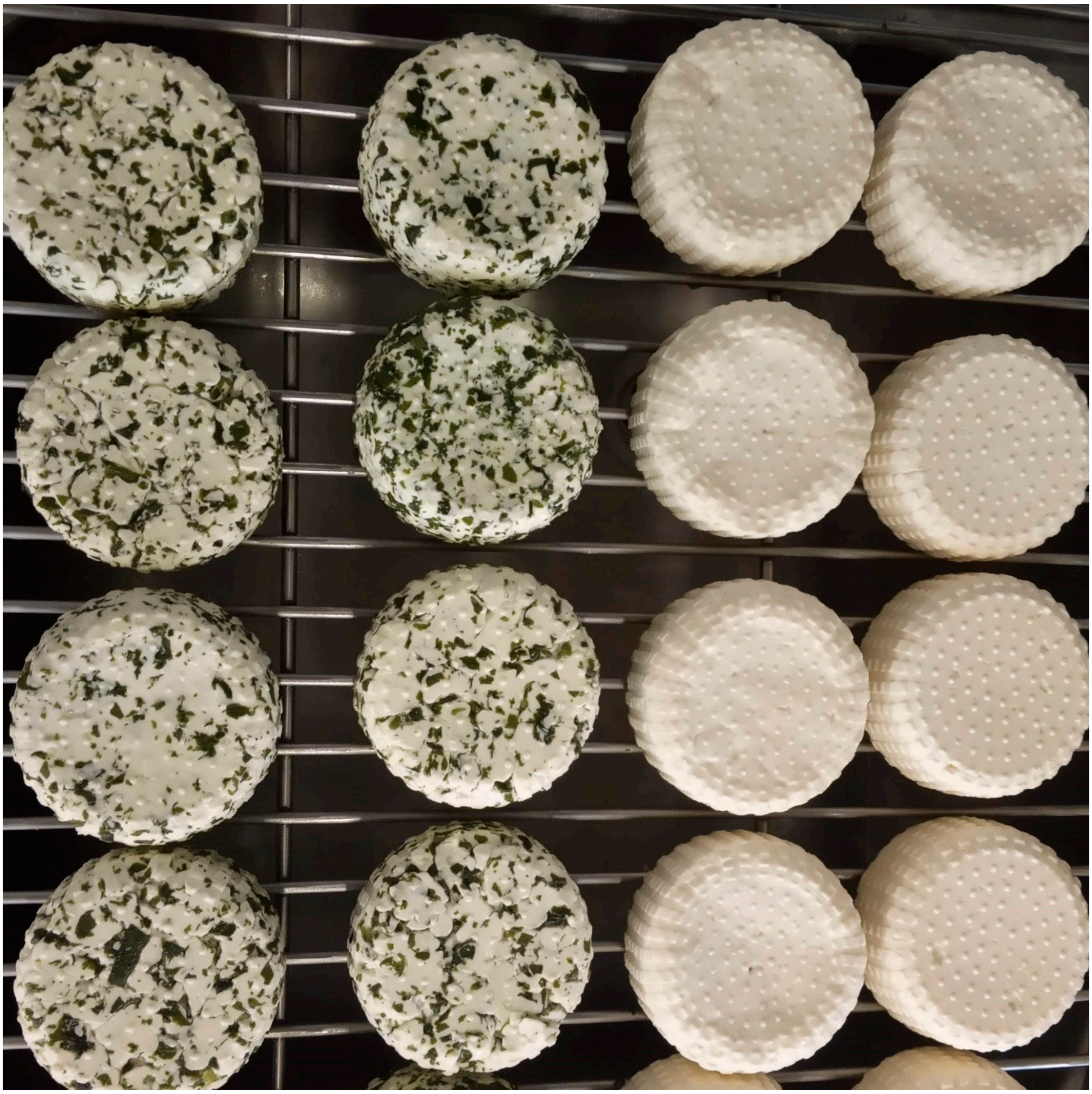

Supplement: Supplementary file 1 [file foods-11-03948-s001.zip › foods-2040825-supplementary.pdf]
